# Just Some Observations on Childhood*Read before the Parent-Teachers’ Association, Santa Barbara, Calif.

**Published:** 1916-05

**Authors:** E. S. Goodhue

**Affiliations:** Hawaii


### Just Some Observations on Childhood.*

BY E. S GOODHUE, A. M., M. D., LL. D., HAWAII.

For the past seven years it has been my privilege to observe and carefully study the physical and mental development of a little girl who came to stay with us that long ago.

I have tried faithfully to eliminate any exaggerated estimate of her intellectual abilities—her special gifts as an artist, musician, logician or philosopher, because I am aware that fond parents with a sort of unconscious egotism, will attribute to their own offspring mental alertness which they never detect in other people's children.

When I was in Boston a few years ago a resident of the city gave a party to some of his intimate friends, and exhibited his son, a boy of six years, who could answer any question in ordinary law; in fact, the lad qualified for the bar before he was seven. To me this precocity is very sad. I look upon it much as I do upon bodily deformity, an abnormal mental development which unfortunately in this case was encouraged instead of being retarded and repressed. And, on discussing the matter at our family table, we expressed our gratitude that our children were commonplace, and in no danger of being ranked as paragons of excellence in any one particular thing.

*Read before the Parent-Teachers' Association, Santa Barbara, Calif.

But we were equally thankful that they were not stupid as some poor children are; that they had the usual number of legs and arms; that their eyes were bright and their ears quick to detect any new thing. In other words, that they were normal children with minds susceptible of cultivation.

And we decided at the council board that these two children of ours should have a chance to grow physically capable before we stimulated their intellectual part to any extent, letting them learn what they might through their senses, taking care that what they did see and hear would be what was good for them.

The boy, who is seventeen, God bless him, did not take to books. We have a large library, and he had all the books he wanted and more,—he built towers and fortresses with them. He is what the psychologists would call a motor-boy, and nothing in the world outside is dull to him. His perceptive faculties developed quickly, he observed closely, "took everything in," and came to his books on nature and physics just to find out something he couldn't learn anywhere else. As he grew older I endeavored to interest him in poetry, in literary forms, the thoughts of authors, but without success. He has more inventions than Edison, is familiar with every mechanical detail of every sort of machine; he can "fix" anything. His collection of butterflies and plants correctly classified are really good, and, without any training, he can draw well. His sketches of animals and men are true to life—his caricatures seem clever. This is all mechanical, technical, but if you ask him to sit down for an hour's reading or study, he looks the picture of distress. It bores him dreadfully; he sighs and wishes he was a sailor, or a pirate down near Borneo.

Of course he and his sister have the same ancestry, and have grown up under conditions and influences very similar, but we have been altogether unable to interest him in literary matters; he says that he is going to be a bacteriologist and "find out things" for himself, but he wants to bridge the study period—"cut it out," he says—and begin being a practical scientist at once.

Now, little sister is entirely different. We haven't given so much time to her as we did to her brother, as there were other things to do, and perhaps we were a trifle discouraged, or, at least, doubtful of the wisdom of our methods. So she was left to grow and reach out without very much suggestion from us, until her fourth year. Then I read to her suitable stories, made some up for her, allowed her to memorize bits of Stevenson or Field. She seemed fascinated by them, and when our reading hour was over, took a book and "read" for herself, voluble stretches of fancy which generally we had to bring to a close.

To her books, as books, are sacred; she fondles them, looks at their bindings, print, illustrations, and is really a good judge of the material aspects of a volume. She plays writing novels, keeps a printing office and bindery and when visitors come, asks them what they have read. Not a day passes that she does not go to the library and handle all sorts of volumes, remarking upon their titles and inquiring about their contents. We could not any more cure her of this propensity than we could her brother of his desire to be "doin' somethin'." Nor have we in any respect been elated over this tendency, or on any occasion allowed the child to "show off" before company, but rather discouraged an excess of development in that direction. So we have here an evidence of the *elan* or push of inherent tendencies, as well as the effect of environment upon two different temperaments.

We have taught sister nothing out of books, except to answer questions and to read and explain where the sense was not clear. Brother rarely refers to the dictionary—sister is anxious for an excuse to do so, and is already handy with the "Century," and reference books of all kinds. She has learned to read and write despite us, has picked up some arithmetic, French, and other bits of learning in her wanderings in the library and class-room, where poor brother sits in misery. Like all children, sister is passionately fond of flowers, the fields, the garden, her pets, but she finally uses them all in her literary compositions, singing her poetry as she trips along.

The day she was three years old she came to me where I was reading, took my hand and asked me to go to the west veranda and look at the sunset. Then she looked up wistfully and said: "Isn't it beautiful, papa?" "Yes, dearie," I answered. "I would like to be up there," she continued, her face radiant with anticipation. "But, dearie," I protested, "you wouldn't be near papa then." And quick as a flash—"but I would be near to God." Here was an indication of ideality which I did not expect to see so early, due, possibly, to her association largely with us, as we generally make a good deal of the sunsets. They are often very beautiful thrown against the western sky with the great ocean for a staging, and give one a sense of infinitude. Sully says, "that children often appear to the adult as unfeeling as a stone, is, I suppose incontestable," and La Fontaine spoke of the age of childhood as "pitiless."

I have noticed in my children, sometimes, a want of consideration for the feelings of others which an ill-mannered adult may show, and a manifestation of selfish thoughtlessness perhaps peculiar to immaturity, but I have not discovered in them any want of sympathy or pity for suffering which they understand and can apply to themselves. In this they are not different from adults, for under certain conditions there is no pity in the matured man or woman. And unless the person suffering is allied to us by close ties of common life and feeling, we cannot really sympathize with him. Our sorrow for ten thousand stricken men and women in India is not acute, although we pity them in a Christian way, and do bear a form of grief, yet it is true that our tears cannot flow as they certainly did over the story of a little child we read the other day. In one instance there is no basis for appeal, in the other there is.

And it is the same with children—they must reach a certain stage of experience and development before they can feel deeply for the sorrows of others; sorrows which have come, or they realize may come, to them. Therefore, I do not think that it is correctly putting it to say that children are pitiless, hard; that they are barbarians and savages. When children are really bereaved by the loss of a pet dog or cat, they show a deep sorrow and sympathy which is often as disinterested as any grief we feel.

When brother was five he lost a little dog. Pupsie died. He grieved over it more than I thought was good for him, and finally when his tears were dried, he said to me: "Pupsie went to sleep, papa. He will n-e-v-e-r, never wake up. He died while he was asleep and didn't know anything about it. And he will go *up*, papa, up to God. Poor little doggie (a world of pathos in this), asleep, never to wake up. God will take him. God made little Pupsie, God is the king of us all—he owns us all. God sent me to you, didn't he, papa? He answers good little boys' prayers, doesn't He?" Then after quite a silence, "Am I a good little boy, papa? Well, I prayed for poor little Pupsie," and in a moment my little lad was asleep.

It is easy here to trace the course of reasoning, and to discern the shades of sorrow in the boy's mind, and his final faith in the goodness of God.

Some evenings after this while sitting on my knee, he looked up at a picture of my mother, and said with a sigh: "She is dead, like Pupsie, papa. If she wasn't I would write to her. She is looking down from heaven, papa, and she says to the angels,

'There is the one who used to be my *own* little boy.'" He had thought it all over. The sharpness of grief had disappeared, but he was lonely and thought of his grandmother who, if she were alive, would no doubt sympathize with him as she so gently could. He would like to write it all down, as we do our sorrows in one way or another.

I do not believe that what the scientists say of the boy can be universally applied—"the world he lives in is separate and distinct from the world the man lives in—the boy is a savage and a barbarian." I do not think that I have (at the same age) noticed as much evidence of deep feeling in sister, although she has had sorrows over the loss of her rabbit, and "pussy," and gave them elaborate funerals. Her self-pity was greater, I think. Perhaps that is feminine, though I shall not undertake to say that it is.

Brother has always shown a "grown-up" sense of sorrow, a sympathy and real concern for any form of suffering which I do not think I possessed when I was his age. He does not like to kill or see things killed; I used to glory in butchering when I fancied each squealing pig was a prisoner taken out of the Conciergerie and brought to the guillotine! What romance there was in it! Perhaps the boy has less fancy and imagination which, in sister, aids her in bearing her sorrows lightly.

There are so many tender things about childhood which, if we are unknowing or careless, we may trample on and destroy. I have often realized with Dr. Bridge that "education should come unconsciously like the learning of the games and child fashions from playmates. The growth of the mind should be as free from any indication of shock or strain as are the wholesome activity and unfoldings of the years of infancy, and young childhood before the education is begun to be thought of," and I stand in fear of—"the watchful zeal of all the self-constituted and often nagging monitors, who stand over us to see that we do not go astray." As Sully says, "So far from saying that child nature is utterly bad, or beautifully perfect, we should say that it is a disorderly jumble of impulses, each pushing itself upward in lively contest with the others, some towards what is bad, others towards what is good. It is out of this motley group of tendencies that the hand of the cultivator has to work, selecting, arranging, organizing into a beautiful whole."

(To be continued.)

---

Dr. O. L. Thweatt and family, of Vashti, have moved to Post.

